# Right hepatectomy in absence of the left portal vein using the porto-rex shunt procedure

**DOI:** 10.1515/iss-2024-0008

**Published:** 2024-08-09

**Authors:** Sven A. Lang, Jan Bednarsch, Sophia M. Schmitz, Marius J. Helmedag, Iakovos Amygdalos, Daniel Heise, Maxime Dewulf, Tom F. Ulmer, Ulf P. Neumann

**Affiliations:** Department of Surgery and Transplantation, 39081 University Hospital RWTH Aachen, Aachen, Germany; Department of General, Visceral and Transplant Surgery, 39081 University Hospital Essen, Essen, Germany; Department of Surgery, 39081 Maastricht University Medical Centre (MUMC), Maastricht, Netherlands

**Keywords:** portal vein anatomy, porto-rex shunt, surgical technique, liver resection

## Abstract

**Objectives:**

Exact knowledge of the portal vein (PV) anatomy is essential for any hepatobiliary procedure. Absence of the portal bifurcation with the complete blood flow to the left lobe coming from the right portal vein (RPV) is an extremely rare anatomical variation.

**Case presentation:**

In this situation, a solitary metachronous colorectal liver metastasis with suspected infiltration of the RPV and the right bile duct was detected in a 51-year-old male patient. Neither percutaneous ablation nor stereotactic radiotherapy were considered indicated due to the close proximity to the central structures. Hence, a surgical two-step procedure was scheduled. First, a porto-rex shunt with an 8 mm PTFE graft to maintain the portal blood flow to the left lobe was performed. In addition, the RPV was ligated during the same procedure. After recovery, the procedure was completed with a right hepatectomy. The final pathological report confirmed invasion of the right bile duct and the RPV and resection margins were tumor-free.

**Conclusion:**

This case shows that careful preoperative assessment of vascular anatomy is critical. The use of the porto-rex shunt allowed a potentially curative resection in an otherwise irresectable situation.

## Introduction

The portal vein (PV) collects blood from the superior mesenteric vein (SMV) and the splenic vein (SV) and, thereby, is responsible for about 80 % of the blood inflow to the liver. Classically, the main PV divides into a left and a right portal branch (left portal vein (LPV), right portal vein (RPV)) at the portal bifurcation. Further, the RPV subdivides into the right anterior portal vein (RAPV) and the right posterior portal vein (RPPV) to segments V/VIII and VI/VII, respectively. This PV branching is found in 65–80 % of cases and, therefore, is generally regarded as normal (type I). In the remaining 20–35 % of cases, multiple variants of PV branching mainly depending on variants of the RPV have been described, with trifurcation (type II) and RAPV arising from the LPV (type III) being the most common [1].

The portal bifurcation finds its origin in the left umbilical vein and the vitelline vein during the embryological development. Absence of the portal bifurcation is therefore considered to be a failure to establish the communication between these two vessels. In cases with non-bifurcating PV, the LPV arises from the right side and follows an intrahepatic course. Thereby, the whole portal blood flow to the left liver is maintained via the RPV and the RAPV, respectively. This variation of the PV anatomy was first described by Couinaud in 1957 and since then only a handful of cases have been published reporting this anomaly [2]. In 2021 Terasaki and coworkers analyzed multidetector computed tomography (MDCT) scans from more than 17,500 patients and found a non-bifurcating PV in only five of them, which equals less than 0.03 % [3]. Hence, absence of the portal vein bifurcation is an extremely rare but nonetheless important variation of the PV.

Surgical procedures, particularly major liver resection can be challenging when the PV is non-bifurcating. While left hepatectomies are easy to perform due to the missing LPV, right hepatectomies are dangerous due to the missing portal blood flow to the left side when the RPV is transected. Therefore, optimal preoperative planning is mandatory to prevent disastrous complications. The current case report describes a patient with non-bifurcating PV who presented with a central colorectal metastasis in the right liver lobe. Due to the proximity or even infiltration of the RPV and the right bile duct, right hepatectomy was necessary to obtain tumor clearance. We herein provide our technical solution for this tricky situation by performing a two-step approach with initial porto-rex shunt and subsequent formal right hepatectomy.

## Case presentation

**Figure 1:**
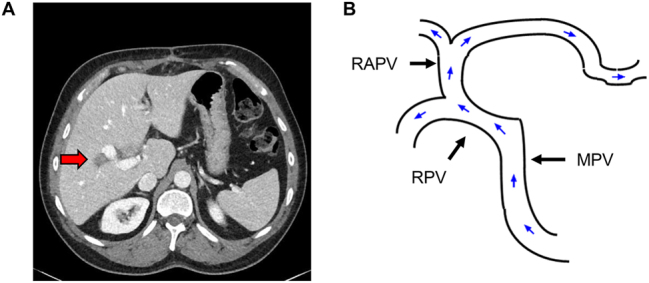
Preoperative status. (A) Central location of the metastasis at the right anterior portal branch (red arrow). (B) Schematic presentation of portal venous blood flow (MPV, main portal vein; RPV, right portal vein; RAPV, right anterior portal vein).

**Figure 2:**
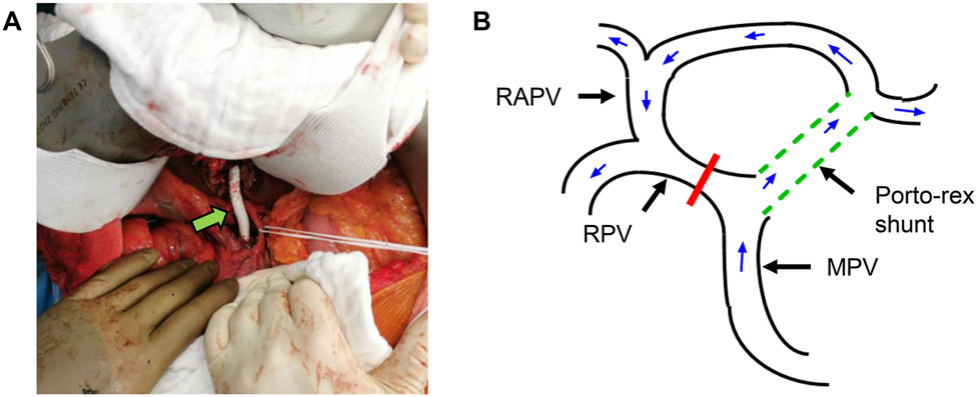
Intraoperative picture and status after the first operation. (A) Porto-rex shunt with PTFE prosthesis during the first operation (green arrow). (B) Schematic presentation of portal venous blood flow via porto-rex shunt (dotted green line) at the end of the first operation. The RPV is ligated (red bar; MPV, main portal vein; RPV, right portal vein; RAPV, right anterior portal vein).

**Figure 3:**
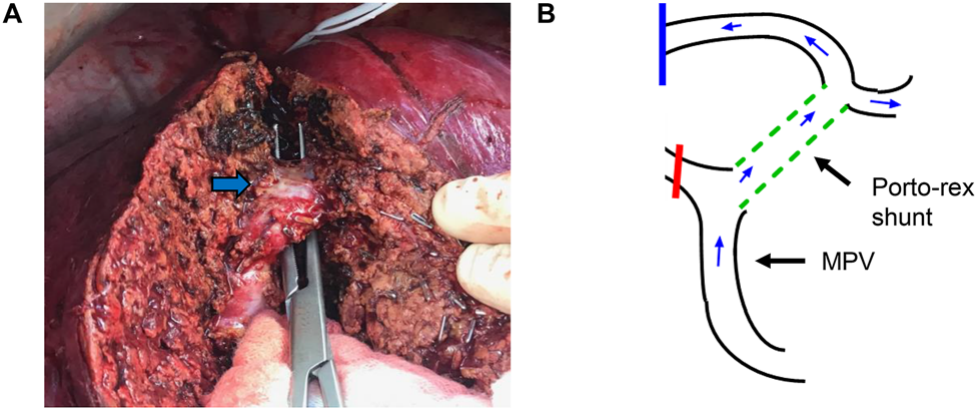
Situs during the second operation and postoperative status. (A) Intrahepatic dissection of the portal vein between the left and the right liver lobe during right hepatectomy (blue arrow indicates portal vein). (B) Schematic presentation of portal venous blood flow via porto-rex shunt (dotted green line) after right hepatectomy. The red bar indicates the stump of the RPV, the blue bar indicates the intrahepatic stump of the portal vein (MPV, main portal vein; RPV, right portal vein).

A 51-year-old male patient with a history of rectal cancer was referred to our center due to a metachronous liver metastasis in the right liver lobe between the RAPV and the RPPV (Figure 1A). The patient had undergone explorative laparotomy in an external hospital with the intent to remove the lesion. During the intraoperative assessment, the abnormal portal vein anatomy was recognized and the operation was not continued at this point. MDCT scan revealed the absence of the portal bifurcation. The complete portal blood flow to the left liver arose from the RAPV and followed an intrahepatic course (Figure 1B; Supplementary File 1 (Video 1)). However, the left liver did not show any sign of parenchymal agenesis. Due to the central location of the metastasis and the suspected infiltration into the RPV and the right bile duct, parenchymal sparing liver resection was not possible. Furthermore, local ablative therapy and stereotactic radiation were discussed but rejected due to the close proximity of the central structures. Hence, we were looking for a surgical solution for this problem and decided to go for a two-step approach. First, a porto-rex shunt procedure with 8 mm PTFE prosthesis and simultaneous ligation of the RPV was performed during the initial operation (Figure 2A and B). Portal perfusion via the porto-rex shunt was confirmed by ultrasound during the operation and by MDCT scan postoperatively (Supplementary File 2 (Video 2)). After recovering, an anatomic right hepatectomy was performed. The original portal vein to the left side was dissected deep inside the liver parenchyma (Figure 3A and B). Sufficient portal perfusion via the rex shunt was again determined by ultrasound during the operation and subsequently confirmed by MDCT (Supplementary File 3 (Video 3)). The postoperative course was uneventful and the pathological report confirmed the metastasis infiltration into the right bile duct and the right portal branch. Surgical margins were tumor-free (R0 resection). The postoperative recovery was uneventful. The patient was doing well and without signs of recurrence for more than 3 years. Forty-two months after the initial liver operation, a single recurrence was detected in the left lateral sector of the liver that was successfully treated by re-resection. The patient is now doing well 48 months after the first liver resection, without signs of further recurrence.

## Discussion

Several anatomic variations of the PV have been described and several classifications have been published so far. In particular, three to five patterns of portal vein branching have been defined, depending on variations of the right-sided portal branches [1]. However, the current case with complete absence of the portal bifurcation and the intrahepatic course of the LPV is not included in these classifications. Indeed, only a handful of case reports have been published with such an abnormal PV variant [4], [5], [6], [7], [8], [9]. Recently, the rare frequency of non-bifurcating PV was confirmed by Terasaki and coworkers who analyzed a large cohort of patients and detected this anomaly in only 0.03 % [3]. The particular clinical importance of this variation is illustrated by a case report from Koh and coworkers. An unrecognized absence of the LPV resulted in complete PV thrombosis, multi-organ failure and finally death of the patient after right hepatectomy [4]. Similar, Teraoku et al. described a portal vein thrombosis after right resection. However, this patient was successfully treated by anti-coagulation. Nonetheless, these examples highlight that exact knowledge of PV anatomy is essential for any hepatobiliary procedure.

Particularly right hepatectomy is challenging in the absence of the portal bifurcation. Nonetheless, a few reports have been published on this. Two cases published by Lee et al. and Spampinato et al. demonstrate that successful right and also extended right resection are feasible even in patients with a non-bifurcating PV [6], 7]. In both cases, preservation of the portal vein via transection along the trunk and maintaining the intrahepatic course of the LPV was described. In our case, preservation of the portal vein was not possible due to the close proximity of the metastasis to the PV and the bile duct on the right side. Notably, infiltration of both structures was finally confirmed by postoperative histological assessment.

To address this issue, we decided to apply a porto-rex shunt procedure to secure the portal blood flow to the left liver. The porto-rex shunt (also rex-shunt or mesenterico-portal shunt) has initially been described to treat PV thrombosis after pediatric liver transplantation. It was subsequently used to treat portal hypertension in children with PV thrombosis and otherwise healthy liver. In adults this procedure has rarely been used so far. Only a few cases have been published using the porto-rex-shunt in patients with advanced extrahepatic biliary tract cancer. Recently, Conticchio et al. reported a series of 11 patients who underwent porto-rex shunt and subsequent hepatectomy with acceptable perioperative results and long-term outcome [10]. However, using the porto-rex shunt to maintain the portal perfusion of the left liver in the absence of the portal bifurcation has not been described before.

A debated issue is the type and size of the interposition graft to be used for the porto-rex shunt. In our case we decided to use an 8 mm PTFE ring reinforced prosthesis for two reasons: first and foremost, the stability of the prosthesis is assumed to be better in preventing any kinking compared, to autologous vein interposition, and, second, to avoid any additional morbidity of vein harvesting. Regarding the size of the prosthesis, Conticchio et al. reported that an 8 mm prosthesis was used for the first patient in their series, who subsequently suffered from ascites. Therefore, a median diameter of 12 mm was subsequently used for the following patients [10]. Since our patient did not show any postoperative ascites, we conclude that the 8 mm diameter was sufficient. Naturally, this decision should be made on an individual basis.

Performing the surgery as a two-step procedure might be a point of criticism. We decided to go for this staged approach to be sure that our PTFE prosthesis remained patent on the long-term. Although intraoperative ultrasound confirmed patency of the shunt, a thrombosis in the early postoperative course would have been fatal in case of concomitant right hepatectomy. In addition, we aimed to avoid any contamination of the PTFE graft e.g. due to postoperative bile leakage. During the second operation, the PTFE graft was completely covered with scar tissue, making a contamination in the postoperative course less likely.

Finally, one might argue that other potentially curative treatment options, such as local ablation or systemic therapy could have been an alternative for this patient. However, due to the central localization of the metastasis, interventional ablation or irradiation bears a high risk of damage to the PV and the bile duct. Furthermore, ablation in close proximity to the PV has a high chance of being incomplete due to the heat sink effect of the major vessels. Systemic chemotherapy as the last option can lead to complete (radiological) remission in some cases. Nonetheless, this effect is uncertain and even in the case of complete disappearance of the metastasis upon radiological imaging, viable tumor cells are still detectable in 30–60 % of cases, with a high risk of recurrence. Therefore, surgical resection was considered the most effective curative treatment in this young patient.

## Conclusions

Careful assessment of the vascular anatomy of the liver is critical for surgeons performing major liver surgery. Absence of the PV bifurcation is a very rare but nonetheless extremely important variation and establishment of a porto-rex shunt provides a technical solution of this problem, when right hepatectomy is required. In our case, this strategy offered a potentially curative option for a patient who otherwise would have undergone palliative therapy. The latter might further underline the referral of patients with liver metastases that are initially judged as irresectable to specialized centers.
